# Intranasal adminsitration of oxytocin in postnatal depression: implications for psychodynamic psychotherapy from a randomized double-blind pilot study

**DOI:** 10.3389/fpsyg.2015.00426

**Published:** 2015-04-20

**Authors:** Andrea Clarici, Sandra Pellizzoni, Secondo Guaschino, Salvatore Alberico, Stefano Bembich, Rosella Giuliani, Antonia Short, Giuseppina Guarino, Jaak Panksepp

**Affiliations:** ^1^Psychiatric Clinic, Department of Medical, Surgical and Health Science, University of TriesteTrieste, Italy; ^2^Institute for Maternal and Child Health, IRCCS “Burlo Garofolo," TriesteItaly; ^3^NPSA Italian Editorial Consultant, University of TriesteTrieste, Italy; ^4^Department of Integrative Physiology and Neuroscience, College of Veterinary Medicine, Washington State UniversityPullman, WA, USA

**Keywords:** postnatal depression, psychoanalytic therapy, oxytocin, emotion regulation, double-blind method

## Abstract

Oxytocin is a neuropeptide that is active in the central nervous system and is generally considered to be involved in prosocial behaviors and feelings. In light of its documented positive effect on maternal behavior, we designed a study to ascertain whether oxytocin exerts any therapeutic effects on depressive symptoms in women affected by maternal postnatal depression. A group of 16 mothers were recruited in a randomized double-blind study: the women agreed to take part in a brief course of psychoanalytic psychotherapy (12 sessions, once a week) while also being administered, during the 12-weeks period, a daily dose of intranasal oxytocin (or a placebo). The pre-treatment evaluation also included a personality assessment of the major primary-process emotional command systems described by Panksepp () and a semi-quantitative assessment by the therapist of the mother’s depressive symptoms and of her personality. No significant effect on depressive symptomatology was found following the administration of oxytocin (as compared to a placebo) during the period of psychotherapy. Nevertheless, a personality trait evaluation of the mothers, conducted in our overall sample group, showed a decrease in the narcissistic trait only within the group who took oxytocin. The depressive (dysphoric) trait was in fact significantly affected by psychotherapy (this effect was only present in the placebo group so it may reflect a positive placebo effect enhancing the favorable influence of psychotherapy on depressive symptoms) but not in the presence of oxytocin. Therefore, the neuropeptide would appear to play some role in the modulation of cerebral functions involved in the self-centered (narcissistic) dimension of the suffering that can occur with postnatal depression. Based on these results, there was support for our hypothesis that what is generally defined as *postnatal depression* may include disturbances of narcissistic affective balance, and oxytocin supplementation can counteract that type of affective disturbance. The resulting improvements in well-being, reflected in better self-centering in post-partuent mothers, may in turn facilitate better interpersonal acceptance of (and interactions with) the child and thereby, improved recognition of the child’s needs.

## Introduction

The specific aim of this study was to evaluate whether intranasal oxytocin could ameliorate postpartum depressive symptoms, and how such a treatment would interact with concurrent psychotherapy. The more general aim was premised on the recognition that the associated fields of affective neuroscience and dynamic psychology need to be integrated as complementary disciplines that seek to bridge neurodynamic affective brain issues and psychoanalytically inspired concepts about how the mind works (e.g., Kaplan-Solms and Solms, 2000). Our approach was premised on the likelihood that these two associated approaches could be integrated to facilitate clinical practice.

Dynamic psychology, in this context, can be regarded as a field that incorporates all the different streams of thought stemming from psychoanalysis, which all have an emphasis on subjective experience. Psychoanalytic thinking implies, within its theoretical background, that all observable behaviors are derived from a dynamic compromise between different aims (affects and drives), along with developmentally established cognitive structures, some of which may be conscious and some unconscious. Yovell (2013, also see Panksepp and Yovell, 2014) has shown, by bringing psychoanalytic concepts to an experimental therapeutic setting – in this case the use of low-doses of buprenorphine for management of suicidality – that important therapeutic contributions can be made in a rigorous empirical way. Similarly, in this study, we began with, Freud’s (1917) considerations that depression is largely a consequence of positive attachment to other (objects) and to their loss (Watt and Panksepp, 2009).

Another view (Sandler and Joffe, 1965) considers depression to be primarily a basic biopsychological reaction connected to psychic pain *within* the *self* (rather than related simply to object loss). In this latter hypothesis, postpartum depressive symptoms would be an expression of narcissistic imbalances, especially as they are centered on a discrepancy between the *actual self* and an *ideal* (wished for, but unactualized) *self*. Based on these considerations, postpartum depression could arise from a defensive homeostatic reaction to mental pain evoked by this narcissistic conflict, rather than directly to a mourning process related to object loss (Sandler et al., 1963). This was the main *a priori* hypothesis that we wanted to relate to the potential effects of exogenous oxytocin’s contribution to alleviation of depressive symptomatology.

Specifically, our view was as follows: a newly born baby imposes on the mother a sizeable burden in terms of confronting her ideal of how she would like to be as a good mother. This desire may be in conflict with what she has experienced as a child in her relationship with her own mother, a relationship that, ultimately, constituted the bases of her own actual self. It can be noted that even young monkeys brought up in the midst of stressful family environments (e.g., mothers under high and low foraging environmental demands) exhibit sustained differences in social engagements and dominance styles, with offspring of the more stressed mothers exhibiting sustained deficits (Andrews and Rosenblum, 1994). The vicissitudes of such complex conflictual processes can also influence mothers’ affective homeostasis: specifically, such stressful effects may be manifested in mother’s reduced self-esteem, especially in terms of diminished narcissistic “refueling” by the ideal self and the resulting structuring of maternal sense of well-being and safety (Sandler, 1960).

Thus, our model of postnatal depression is based on the general hypothesis that such dysphoria is part of a wider constellation of narcissistic disorders, mainly focused on a conflict between different aspects of the self, rather than simply on the object’s attachment or loss. Each mother may be different in terms of the nature of this conflict. Nevertheless, there are some constant factors: first, all human beings basically share the same basic affective-emotional cerebral systems that constitute neuro-functional networks that are homologous across all mammals that have been studied with deep brain stimulation (DBS), including humans (Panksepp, 1998, 2011a,b; LeDoux, 2000); second, the birth of a child imposes similar patterns of conflict on a mother, ranging from the need to exhibit maternal care and devotion to infants to diverse other life demands, even though each mother may then cope with recurring conflicts using different strategies and defense mechanisms, structured via her own specific developmental history (Sander, 1962; Stechler, 2000).

Neuroscientific findings on the role of oxytocin support the hypothesis that this neuropeptide plays a central role in the mother in the regulation and preparation of the complex processes involved in the care of her offspring: oxytocin has clear behavioral and subjective effects (MacDonald et al., 2011, 2013; Mah et al., 2014) and seems important in decreasing self-centered activities in mothers, while increasing confidence and well-being, which can strengthen the concern as well as confidence for handling infant’s needs (Panksepp, 2009; Mah et al., 2013; Veening and Oliver, 2013). Oxytocin is well known to promote prosocial behaviors, especially in the short term following its administration (Bartz et al., 2011; Bakermans-Kranenburg and van I Jzendoorn, 2013) with a variety of reported effects, of which antidepressant effects are most relevant here (for an extensive recent summary see McQuaid et al., 2014). Functional neuroanatomical studies, with both animals and humans, have demonstrated that oxytocin promotes the arousal of the basic emotional systems for maternal CARE while reducing PANIC (or separation-distress) arousal within the brain (Nelson and Panksepp, 1998; Panksepp and Watt, 2011; Eapen et al., 2014), and potentially directly promote positive affect (IsHak et al., 2011).

The psychoanalytic literature suggests that such basic emotions help process the vicissitudes of important developmental processes such as the establishment of a stable sense of *self* and the progressive development of affective attachment processes (in psychoanalytic terms, *internal objects* of affection). Such attachment schema may progressively become internal templates that tend to be perceived as internal sources of feelings of safety, well-being and affective balance. The structure of each individual is thus the result of experience gained during development, and is also molded by experienced as well as unconscious processes within the relational world of the family. Individual personality traits also depend on such affective dynamics, which are engendered through early relationships with significant others (usually people in the immediate biological family) through the satisfaction (or frustration) of primary physiological and psychological affective needs, from birth onward. The relational model of psychological functioning is therefore crucial for understanding the level of maturation of all individuals, as they progress through critical periods of childhood, with an appreciation of the ways in which new mothers have conducted their relations with children throughout the course of their developmental trajectories (Fonagy et al., 1991; Schore, 1994). Reductions in plasma oxytocin have been noted in depressed women (Yuen et al., 2014), especially in women exhibiting post-partum depression (Kim et al., 2014). Indeed, psychosocial stressors (as a child’s birth invariably is) tend to reduce plasma oxytocin more in women who get depressed than those who do not (Zelkowitz et al., 2014).

The birth of a baby, like any new major significant change, motivates a thorough revision within the mother of her internal world, both in respect of her objects and, in particular, of her own self-functioning modalities. Her objects may be structured, particularly in deprived individuals, as traumatic or eventually confusing object relations, and may be repeated, re-lived and re-enacted in the current relationship with the newborn child. In this case, we would consider the depressive symptoms to be the consequence of a complex series of events, which starts with a failure of the separation-individuation process and the capacity to mourn, and leads to the unconscious rejection of the lived experience of loss in the mother (Mahler, 1974). This complex series describes a form of depression that arises from a problem in the relationship with the object of loss, as described by Freud (1917) in his famous paper on *Mourning and Melancholia*. This form of depression is usually also called *introjective depression* because its dynamics are focused around the unconscious vicissitudes of object loss, and the defensive mechanism of introjection (Blatt, 1974).

### Hypothesis and Aims of the Study

In psychodynamic thinking, therefore, depression can be seen as a defensive condition against mental pain. We reviewed here, in particular, the hypothesis that depression is a narcissistic response to an intrapsychic conflict (Sandler and Joffe, 1965). In postnatal depression, the origin of the conflict is where the narcissistic needs of the mother meet the drive to attach to the infant (bonding or, in psychoanalytic terms, a conflict between *self cathexis* vs. *object cathexis*). The aim of our study was to evaluate the effects of the neuropeptide oxytocin, particularly when administered in conjunction with a course of introspective supportive psychotherapy. On one hand, basing our hypothesis on Panksepp’s (1998) neuroscientific model of the organization of the basic emotional commands in the brain, we began with the hypothesis that oxytocin has effects on the CNS in stimulating the CARE system (Panksepp, 1998; Zelkowitz et al., 2014), as has been shown in both animal (Insel and Young, 2001) and human studies (Feldman et al., 2007, 2010). Moreover, oxytocin has been shown to have general prosocial effects in healthy (Kosfeld et al., 2005) and pathological (MacDonald and MacDonald, 2010) human subjects. Oxytocin is also known to stimulate the SEEKING system (‘reward’ system, Panksepp, 1998; Bartels and Zeki, 2004), and is involved in a reduction of the activity of both the amygdala (Kirsch et al., 2005) and the hypothalamic-pituitary-adrenal axis (Neumann, 2002). All this implies that oxytocin may have especially soothing and confidence-enhancing effects in stressful social events of high interpersonal value: one such instance is the insurgence of *postnatal depression* affecting the formation of a mother’s bond with her newborn child.

In line with our main hypothesis that postnatal depression is more of a self-centered anaclitic depression, we introduced a psychotherapeutic setting, which is known to influence the processes of depressive pain, administered alongside oxytocin, which is known to facilitate maternal behavior. On this basis, we established an experimental situation into which we introduced a variable that consequentially affects the brain. In our hypothesis, we assumed that postnatal depression arises from an imbalance within basic subcortical systems, with an increase in activity of the system modulating PANIC, namely separation-distress (in psychodynamic terms, a system mediating core self-regulation inducing an anxiety state related to potential stressful longing; Panksepp and Watt, 2011), and a decrease in activity of the system modulating CARE and SEEKING (an emotional system that is more involved in ones approach to others, or – in dynamic terms – in *object cathexis*). The first system is more characterized by negative (avoidance) emotions, and the second more by positive (approaching) emotions (Panksepp and Watt, 2011).

Data from other studies has suggested that long-term administration of oxytocin, rather than having direct anxiolytic or antidepressive effects (as is the case in the natural development of the relationship between caregiver and offspring, in both humans and animals), may serve as the facilitator of a supportive relationship. For this reason, with mothers with mild to severe signs of postnatal depression, we combined a treatment of supportive, introspective, psychoanalytically oriented psychotherapy with the administration of oxytocin, in a randomized double-blind, placebo-controlled study. We concluded that postnatal depression was present when a mother was referred (by any member of the ward staff) due to the signs and the symptoms described below in **Table 1** and when the mother voiced major intrapsychic conflicts of being in need of support and at the same time having an intense expectation of being supportive toward her newborn child, a conflict indicating the mother’s suffering as a result of her inability to create a supportive bond with the newborn child.

**Table 1:** List of Beck’s (2001) criteria for postpartum depression.

Significant predictors for postpartum depression (Beck, 2001)
Prenatal depression
Low self-esteem
Child-care stress
Prenatal anxiety
Life stress
Low social support
Poor marital relationship
Difficult infant temperament
Maternity blues
Single marital status
Unplanned/unwanted pregnancy
Low socioeconomic status
History of previous depression


In order to test these hypotheses, we devised and chose several testing procedures to examine the following questions:

(1) Does intranasal oxytocin administration influence the self-centered aspect (narcissistic *cathexis*) or the investment in the other (the child who needs caring for object *cathexis*) in the relationship between mother and child?
(2) Does oxytocin affect depressive (dysphoric) symptomatology or self-centered painful conflicts?
(3) In what way are basic emotional systems involved?

To evaluate the first hypothesis, we evaluated the mothers using the Shedler–Westen Assessment Procedure (SWAP) scale (Shedler and Westen, 1998; Westen et al., 2008), which has a coherent psychodynamic theoretical basis and provides measures of dysphoric and narcissistic traits. We focused only on the narcissistic and dysphoric traits measured by the SWAP scale, to see whether these variables were influenced by the intranasal administration of oxytocin. According to our hypothesis, oxytocin should have influenced primarily the narcissistic traits in the subjects.

To evaluate the second hypothesis, we relied on two of the most widely used symptomatic clinical scales of depression: the EPDS postnatal depression scale (Cox et al., 1987) and the Hamilton (1967) scale. The first measures the dimension of depression from the more subjective point of view of the mother, and the second from the more objective point of view of the evaluator.

To evaluate the third hypothesis, we tested the mothers before and after the psychotherapy using the Affective Neuroscience Personality Scale (ANPS) (Davies et al., 2003), which measured the mothers’ basic emotional systems and evaluated the differences between groups (mothers in the oxytocin group vs. placebo). In line with our theoretical hypotheses, we expected that certain systems in particular might be involved, such as the CARE system and the PANIC (i.e., Separation Distress) system.

Ultimately, we wanted to evaluate how psychotherapy influences or interacts with the neuropeptide administration: both groups of mothers followed a short-term psychodynamic psychotherapy. We then evaluated the effects of psychotherapy on the two groups to see if there was any interaction between the administration of the neuropeptide and the psychotherapy. We consider this a preliminary study.

## Materials and Methods

### Participants

A sample of 16 mothers was selected from an obstetric ward, after having met at least 4 of the 13 the inclusion criteria listed by Beck (2001) for postnatal depression (see **Table 1**). The mothers had no previous history of neurological or psychiatric disorder. The recruitment of postnatally depressed mothers was carried out by nurses, gynecologists, obstetricians, or pediatricians on the ward, especially if the women were reported as suffering emotional distress following childbirth.

Their age ranged from 29 to 42 years [Mean age: 36.5 (SD 5.6); 11 mothers were primiparous]. The age of their children ranged from 2 weeks to 6 months post delivery [Mean age: 4.5 months (SD 1.2 months)]. Fourteen participants breastfed during the period of therapy, two mothers interrupted the breastfeeding after a few months (**Table 2** shows demographic and clinical data for each mother). Participants had no previous history of neurological or psychiatric disorder, and did not receive any medication.

**Table 2:** Characteristics of the mothers included in the study.

	Age	Nationality	Parity	Breastfeed
**Oxytocin group**
1	41	Ita	2	yes
2	39	Ita	1	yes
3	34	Ita	2	yes
4	38	Ita	1	yes
5	33	Ita	1	yes
**Placebo group**
1	38	Ita	1	1 months
2	33	Outside EU	1	Yes
3	35	Ita	1	Yes
4	41	EU	1	4 months
5	42	Ita	1	Yes
6	38	Ita	2	No
7	38	Outside EU	2	Yes
8	39	Ita	1	Yes
9	38	Ita	1	Yes
10	29	Ita	1	Yes
11	33	Ita	2	Yes

### Experimental Design

The mothers were assessed for symptomatic depression using the Hamilton Rating Scale for Depression (HRSD; Hamilton, 1967) and the Edinburgh Postnatal Depression Scale (EPDS; Cox et al., 1987). They were then randomly assigned, in double-blind fashion, to either medicinal treatment with *Oxytocin* or a *Placebo*. Due to the randomization list, initially planned for 30 participants, there was an unbalanced distribution of participants between groups. The *Oxytocin* group (*n* = 5) self-administered the neuropeptide intranasally, daily in the morning: two sprays (approximately 100 μl each) per nostril, from a spray inhaler of oxytocin containing 40 IU/ml, corresponding to a dose of 16 IU/day (Landgraf, 1985). The second (*Placebo*) group (*n* = 11) of mothers was treated with the administration of a placebo composed of a saline solution self-administered intranasally following the same instructions given to the oxytocin group. Both groups were also referred to a psychological psychodynamic support, once a week for 3 months.

The study protocol (see **Table 3**) was approved by the ethics committee of the local “Burlo Garofolo” Children Hospital, and written informed consent was obtained from participants. This study was registered in the Public Pharmaceutical Observatory of the Italian Health Service Ministry.

**Table 3:** Experimental design and sequence of the assessments.

#	Encounters	General administration	Assessment tools
1st	Referral	Randomization (assignment to the placebo or to the oxytocin group	Beck’s (2001) criteria scale
2nd	Pre-treatment assessment meeting	No substance administration	EPDS, HDRS, ANPS scales
3rd	First psychotherapic encounter	Beginning of the daily administration of substance (either Oxytocin or Placebo)	SWAP scale by the therapist after the first session
4th– 13th	Weekly sessions of psychotherapy (globally 12 sessions)	Continuation of daily administration of substance (either oxytocin or placebo)	Weekly follow-up supervision sessions by the team of psychotherapists
14th	On the 12th Psychotherapic encounter	Continuation of daily administration of substance (either oxytocin or placebo)	SWAP scale by the therapist after the last session
15th	Post-treatment assessment meeting	Discontinuation of daily administration of substance	EPDS, HDRS, ANPS scales

After having met Beck’s (2001) criteria for postnatal depression, all the mothers were randomly assigned either to the Placebo or the Oxytocin group; both groups then underwent a first pre-treatment assessment meeting with an examiner (author S.P.), using as empirical evaluative tools the two tests assessing symptomatic depression (EPDS and HDRS). The first test addressed the subject’s self-perception of depressive symptoms (first-person perspective), while the second addressed the examiner’s perception of the presence of depressive phenomenology in the patient (third-person perspective). The pre-treatment evaluation also included an assessment of the basic emotional command systems (as described by Panksepp, 1998): in particular, we used the ANPS (Davies et al., 2003). The ANPS was used to assess the attitude of the mothers toward a set of primary process emotional feelings (SEEKING, FEAR, ANGER, CARE, SORROW, and PLAYFULNESS) which, estimated the status of six of their psychiatrically relevant basic affective-motivational systems.

Seven psychotherapists took part in the study. They were all trained with a psychodynamic approach and, even if the treatment period only lasted for a short period of time, they worked with an approach related to their type of training. In particular, the patient could speak freely about the topic she chose, and no mention was made of the postpartum depression unless the patient was willing to speak about it – trying to be neutral but at the same time showing an *affective presence* (a concept upheld by Sander, 1962) to the patient, creating an interpersonal space where emotions could be expressed (Stechler, 2000). As this was a short, dynamic psychotherapy treatment, an important part of the psychotherapeutic dialog was devoted to the implicit working-through process on the patient’s part, with relevance to the therapy’s forthcoming conclusion, i.e., looking at emotional issues of separation. All the interventions were then discussed with an external supervisor, and all the therapists took part in the supervision twice a month, along with the ongoing therapies with the mothers. In this way we tried to guarantee procedural homogeneity, while also accommodating the different and peculiar personalities of both patients and therapists.

Following the first session with the patient, each therapist had to complete the SWAP scale (Shedler and Westen, 1998), a scale measuring personality styles in the mother. This scale is derived from psychoanalytic inferences. The therapist had to provisionally complete the SWAP scale based on their first impressions after the first meeting. At the end of the psychotherapy treatment, the therapist had to complete the SWAP scale. According to the main *a priori hypothesis of this study,* a comparison was made between the first and final SWAP tests using the dysphoric and narcissistic dimensions provided by this scale. This test allows the therapist to estimate certain aspects of personality, such as the narcissistic and dysphoric feelings directly related to our hypothesis, both before and after the psychodynamic treatments, providing empirical evaluation of the effects of therapy on depressive symptoms.

Finally, the patient underwent a final meeting for post-treatment assessment with the examiner and to repeat the same three tests (EPDS, HDRS, and ANPS) as in the first pre-treatment session.

### Experimental Measures

We used four different scales for the assessing the influence of the administration of oxytocin versus placebo: (1) the ANPS (Davies et al., 2003), (2) the EPDS scale (Cox et al., 1987), (3) the Hamilton Depression Rating Scale (HDRS; Fava et al., 1982), and (4) the SWAP scale (Westen and Shedler, 2007; Westen et al., 2008).

(1) The ANPS is a self-report inventory devised with the aim of studying and evaluating personality from the perspective of affective neuroscience advanced by Panksepp (1998), who identified six basic innate affective systems: the SEEK, FEAR, ANGER, SADNESS, PLAY, and CARE systems. The scale empirically assess the expressions of each basic emotional system and thus the combination of these fundamental motivational elements of human personality and its variants. The original ANPS structure has been retained and contains a total of 110 items in its present form. It is scored on six scales, referring to the six basic affective systems (SEEK, PLAY, CARE, FEAR, ANGER, and PANIC/Separation-Distress). The ANPS was translated into Italian separately by a research group including the authors (AC) and the subsequent standardization of this Italian version of the ANPS has been published (Pascazio et al., 2015).
(2) The EPDS is a 10-item questionnaire that was developed to identify women who have postnatal depression. Items of the scale correspond to various clinical depression symptoms and suicidal ideation. The distinctive characteristic of this scale is that it measures depressive symptomatology from a subjective (first-person) point of view (see also Benvenuti et al., 1999).
(3) The HDRS is the most widely used clinician-administered depression assessment scale. The distinctive characteristic of this scale is that it measures depressive symptomatology from a third-person (the examiner’s) perspective. The version we used contains 17 items pertaining to symptoms of depression experienced over the patient’s past week.
(4) The SWAP scale is a psychological assessment tool for personality diagnosis and clinical case formulation. The scale is completed by a mental health qualified professional in a treatment or assessment context, and does not depend on the accuracy of information provided by patients. The scale comprises 200 personality-descriptive items, each of which may describe a given patient well (scored 7 by the assessor), somewhat, or not at all (scored 0). Software based scoring algorithms compute and graph different profiles, as *T*-scores, which provide: personality disorder diagnoses and dimensional trait scores. In our study, due to our *a priori* hypothesis on the narcissistic and depressive-anaclitic nature of depression, we concentrated the analyses on the SWAP results showing the narcissistic and the disphoric dimensional styles. The *narcissistic style* in fact reflects a more self-centered attitude on the part of the patient toward the conflict evoked by her new status as mother, while the *dysphoric style* reflects her attitude toward loss and attachment (Shedler and Westen, 2007).

### Data Analysis

Since numbers of participants in our groups were rather small we used the more conservative non-parametrical analyses for statistical comparisons. Specifically, between-group comparisons were made with the Mann–Whitney-*U* test, and comparisons pre- and post-treatment were made using Wilcoxon Signed-Rank Tests.

## Results

When Oxytocin and Placebo groups were compared, by *U* Mann–Whitney test, on EPDS, HDRS, ANPS, SWAP’s *narcissistic style,* and SWAP’s *dysphoric style* scores, *no significant differences* emerged, in either the pre- or post-treatment condition.

**FIGURE 1:**
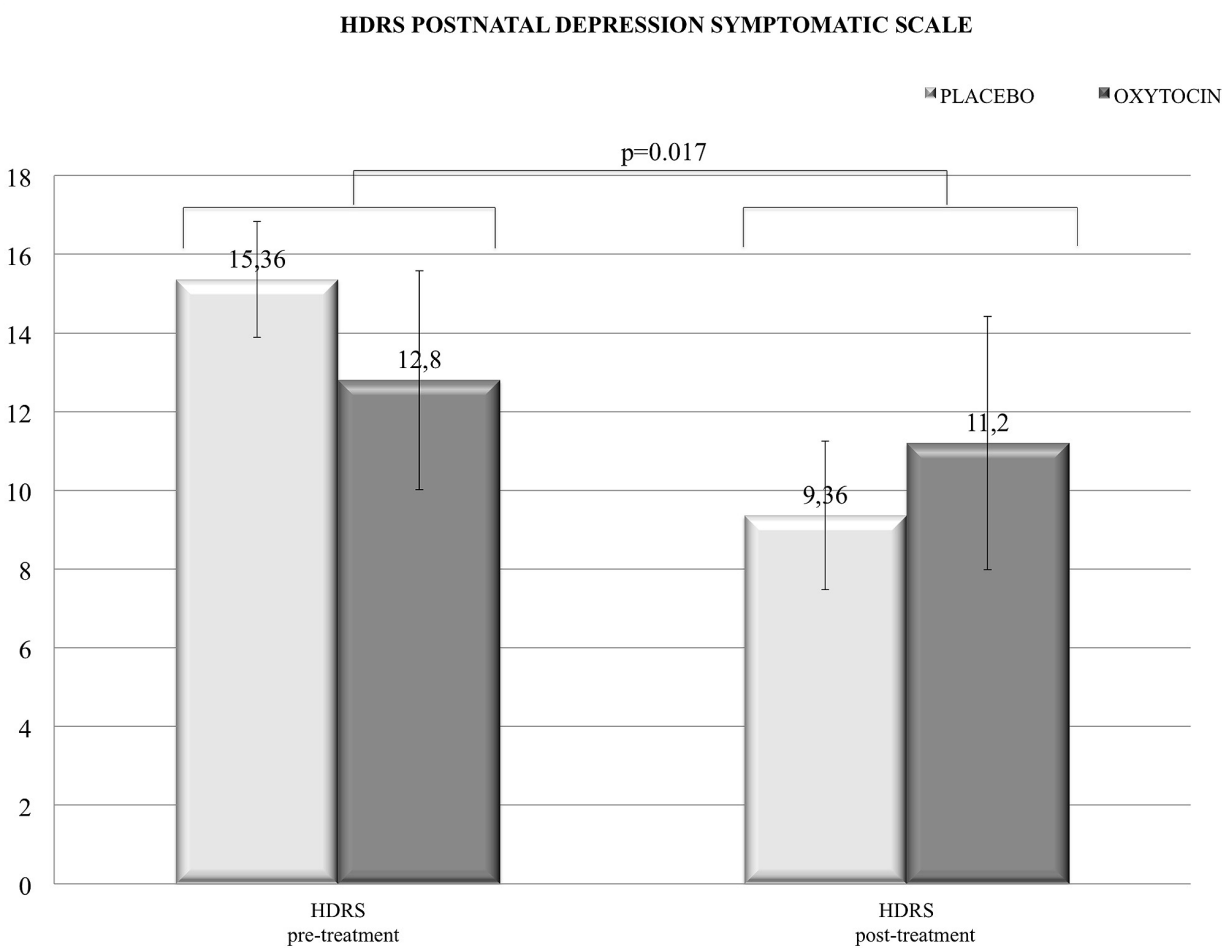
**In the depression symptomatology scale Hamilton Depression Rating Scale (HDRS) we found a positive (decreasing) effect due to the global treatment (psychotherapy), with no clear differential enhancements with intranasal administration of oxytocin**.

When the pre- and post-treatment conditions were compared, separately for each group, by Wilcoxon Signed-Rank test, *no significant differences* emerged on EPDS, HDRS, and ANPS scores in both groups. It has to be noted that the depressive symptoms, as assessed by HDRS, were sensitive only to psychotherapy, e.g., symptoms were globally decreased in the whole sample as revealed by Wilcoxon Signed-Rank test (*z* = -2.355; *p* = 0.017). Thus, there were no specific or direct effects from intranasal oxytocin administration on this variable (**Figure 1**).

**FIGURE 2:**
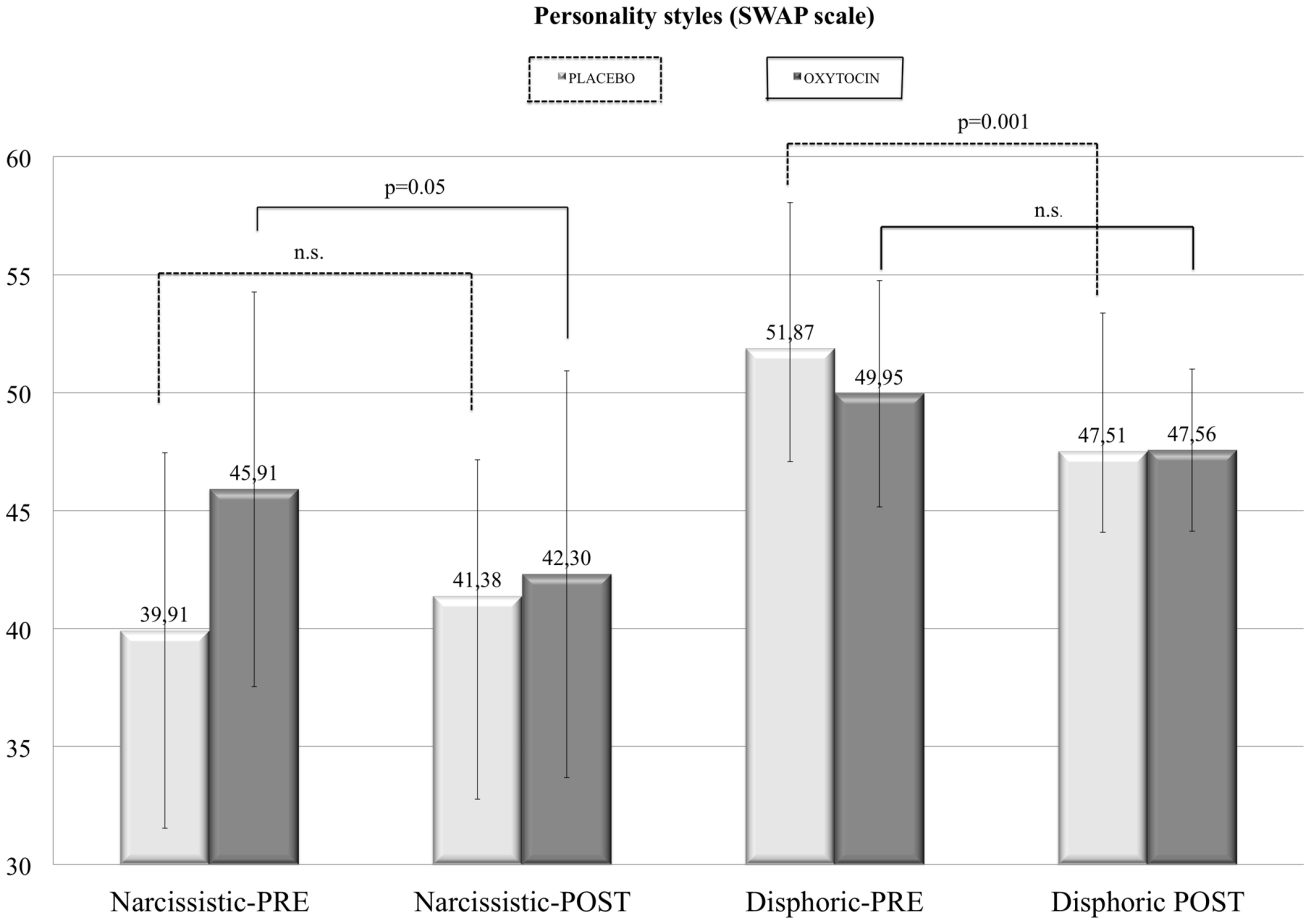
**In the personality style scales Shedler–Westen Assessment Procedure (SWAP) we found a specific (decreasing) effect due to intranasal administration of oxytocin (and psychotherapy), with significant reduction on the narcissistic (self-centered) dimension of mothers with postnatal depression **(left)**, with reductions in dysphoric tendencies in controls but not oxytocin treated mothers (right)**.

In contrast, Wilcoxon Signed-Rank test comparing pre- and post-treatment SWAP’s *narcissistic style* and SWAP’s *dysphoric style* scores revealed significant, although opposite, results in the two groups (**Figure 2**). In the Placebo group, the dysphoric style was significantly diminished (*z* = -3.29; *p* = 0.001) after treatment. In the same group, the narcissistic trait did not significantly change after therapy (placebo plus psychological psychodynamic support). On the other hand, in the Oxytocin group, the dysphoric score was not affected by treatment, while the narcissistic trait score diminished with intranasal oxytocin plus psychological psychodynamic support therapy (*z* = -2.756; *p* = 0.006). When these scores were compared, pre- and post-treatment, across the whole sample, a significant decrease in the *dysphoric style* was observed (*z* = -2.355; *p* = 0.019).

**Table 4** shows the average scores of all independent study variables, and statistical results of comparisons between Placebo and Oxytocin group.

**Table 4:** Average scores from all independent variables considered in this study in the Oxytocin and Placebo groups (results from comparisons between groups were not significant in *U* Mann–Whitney test. The SWAP scores resulted significant using the Wilcoxon Signed-Rank test.).

	EPDS		HDRS		SWAP			


					Narcissistic		Dysphoric	


	Pre	Post	Pre	Post	Pre	Post	Pre	Post
Oxytocin group	15,2	11,8	12,8^a^	11,2^b^	45,9^1^	42,3^1^	50,0	47,6
Placebo group	13,23	9,3	15,4^a^	9,4^b^	40,1	41,7	52,9^2^	48,3^2^
Significance			Δ (a-b): *p* = 0.017		Δ^1^: *p* = 0.001		Δ^2^: *p* = 0.006	

*The ANPS measures are not shown since they proved to be trait measures and were not considered*.

## Discussion

This is the only empirical study, to our knowledge, that has set out to combine the daily administration of oxytocin with short-term psychodynamic psychotherapy. We would emphasize some limitations to our study. First the preliminary nature of these results, since the number of subjects is rather small; however, generally, clinical studies using oxytocin in human subjects range in terms of the number of subjects from single case reports to samples of 40 people. Only non-clinical studies using genetic markers or questionnaires generally have greater samples. Second, we note that there was an uneven distribution of the sample population across the Placebo and Oxytocin groups, which was partly due to the difficulty in convincing mothers (and their doctors) to undergo psychotherapy at such a critical point in the mothers’ lives. To overcome these limitations, we accurately chose more conservative statistical analyses, that allowed the following inferences.

First of all, contrary to our expectations, we found no major effect of oxytocin on overall depressive symptomatology, as compared to placebo (whether observed with the HDRS or EPDS). This is very much in line with the weak effects of oxytocin on depressive symptoms (see MacDonald et al., 2013; Kim et al., 2014). For a full summary of the effects of various treatments on post-partum depression, (see Sockol et al., 2013). However, psychodynamic treatment did have a significant ameliorating effect on post-partum depressive symptoms, as revealed by the significant decrease in HDRS score, but no specific additional synergic effect was found with the intranasal administration of oxytocin. When the experimental groups were considered in statistical analysis, the lack of differential result may be explained: (1) by the small size of both groups and an uneven distribution of participants between them, due to an unbalanced randomization procedure (see above); (2) by time limits: we used a model of short psychoanalytic psychotherapy – 3 months’ worth of sessions – and we could assume that this was insufficient time to robustly address the symptomatic aspect of depression; (3) by our theoretical background, in which depression is a psychobiological reaction or defensive response to mental pain: for these patients, working therapeutically meant, particularly during the first periods of therapy, bringing them closer to their depressive feelings or, in other words, bringing them in closer contact with their conflictual feelings.

Also contrary to our expectations, no differential effects as regards the various basic emotional traits as monitored with the ANPS (Davies et al., 2003; Davies and Panksepp, 2011) were found in the mothers who took oxytocin compared with those who took a placebo: it thus seems that neither our self-centered hypothesis based on the PANIC/Separation-Distress system nor the object-centered hypothesis yielded any clear supportive evidence. Indeed, the overall CARE score (indicating a stable behavioral trait related to maternity, attachment, and object loss) was not significantly influenced by treatment with either psychological psychodynamic support or oxytocin. Thus the ANPS scores should be more appropriately used as “trait” measures instead of “state” measures. Therefore, ANPS scores did not allow to draw any conclusions as regards the treatment with oxytocin.

More decisive analyses were conducted on the psychotherapists’ assessments of the individual mothers. These were accomplished using the SWAP scores in a double-blind procedure. Across the board, in both groups, the dysphoric style decreased significantly. The narcissistic style was not significantly affected by the treatment with placebo. On the other hand, the placebo (combined with the psychotherapeutic treatment) was shown to be effective in decreasing the dysphoric state in the mother, according to the therapists’ observations when comparing their first impressions with their final assessment using the SWAP scale following the overall 3-month treatment period.

Overall, we found that medium-to-long-term oxytocin administration combined with psychotherapy does not seem to affect symptomatic depression, but it does more subtly affect the depressive presentation of the mothers: the therapists double-blindly perceived the mothers as being still depressed but less self-centered (or showing a less narcissistic pattern of behavior with their therapist and possibly with their child). Therefore, oxytocin may ameliorate the narcissistic (self-centered) dimension of the sufferings present in postnatal depression, rather than depressive symptoms *per se*.

In conclusion, this double-blind study serves to highlight that oxytocin may decrease the narcissistic attitude of depressed mothers, especially those relating to the mother’s depressive narcissistic reactions to her intra-psychic conflict. The narcissistic attitude decreased in the mothers undergoing short-term psychotherapy plus oxytocin, as compared to the mothers undergoing only psychotherapy while receiving placebos.

These results are in line with the hypothesis that oxytocin enhances prosocial behavior, perhaps by relieving the suffering of the mothers (as found in some other studies but not in ours), especially (as in our study) through the facilitation and induction of a greater tolerance of self-object differentiation. This effect is more evident if the emotional configuration of the postnatally depressed mother is taken into account, in particular their baseline caring disposition in relation to others (in general) and toward the newborn in particular. Preventive measures could be taken as a result of studies like this (if the results are confirmed) to help both mothers in distress and newborns during this critical period involving the formation of a new bond, a period in a child’s life that tends to affect their entire life cycle (for discussion, see Stechler, 2000).

In our study, the administration of oxytocin particularly affected the self-centered (narcissistic) dimension of postnatal depression. Specifically, following treatment in the group of mothers who took oxytocin, narcissistic aspects appear to have decreased. This data is consistent with the theoretical hypotheses presented above, showing that postnatal depression may be attributed to the wider category of disturbances of the narcissistic homeostasis. Such shifts in maternal moods throw up abundant implications for the psychologically healthy secure development of infants (see Stechler, 2000).

These results confirm another way of understanding depression, which is more centered on a narcissistic disorder, a disturbance within the *self*. When, in the course of development, an individual – a mother, in our case – has not structured a sense of self sufficiently separated from her object (or when she has defensively distorted her objects as being non-caring), she cannot form a reliable internal bond (or object-relation) on which to depend, and the consequence is an *anaclitic depression* (Blatt and Bers, 1993). Freud (1914) addressed this issue in his famous study on narcissism where he proposed that any person – in our case a mother – may love according to (a) the *anaclitic (attachment) type* of object choice (based on the woman who once fed her, or on the man who protected her, i.e., on the operational models introjected by her parents), or (b) the *narcissistic type of object choice*, which seeks the subject’s own self and finds it again in significant others (choosing herself as an object of cathexis, usually a lost part of herself, or an ideal part of herself, i.e., what she would like to be, or investing these attributes into someone who was once actually part of herself, her newborn child; see also the footnote added in Freud, 1905, p. 222). In the latter case, the maternal conflict arising in the postanatal period is of a narcissistic kind: the mother’s desired image of the self does not coincide with her actual feelings; a sense of general inadequacy is present at a time in her life when her resources are already under stress following the delivery of her child. This cognitive and affective discrepancy is a major source of psychic pain, and in this case depression may be viewed as an effective general (psychobiological) defense mechanism to dampen the intensity of an intolerable pain. Therefore, generally speaking, we support the hypothesis that postnatal depression is more often characterized by this deep internal dynamic: the newborn child imposes on the mother an urge to respond to demands within the self (rather than depending on her relationship with her objects), and if these demands were previously unmet within a deprived or over-dependent mother, that mother may experience feelings of inadequacy as a result of this unresolved painful conflict. Inevitably, the mother’s developmental failures combine with the development of her child’s dependency issues, increasing the mother’s intolerance and carrying her into the vicious circle of postnatal depression. Despite several limitations, this pilot study seems promising in certain respects: in general, it points in the same direction anticipated by previous human studies with oxytocin, and it suggests that ideas resulting from theories based on the idiographic tradition of psychoanalytic theory may serve as an inspiration for further, more comprehensive standardized experimental studies.

## Conflict of Interest Statement

The authors declare that the research was conducted in the absence of any commercial or financial relationships that could be construed as a potential conflict of interest.
